# Differential Expression Profiles of mRNA and Noncoding RNA and Analysis of Competitive Endogenous RNA Regulatory Networks in Nonalcoholic Steatohepatitis

**DOI:** 10.1155/2022/3200932

**Published:** 2022-07-07

**Authors:** Mengjia Gao, Jingxin Xin, Xiaoling Li, Ling Gao, Shanshan Shao, Meng Zhao

**Affiliations:** ^1^Department of Endocrinology, Shandong Provincial Hospital Affiliated to Shandong First Medical University, Jinan, Shandong 250021, China; ^2^Shandong Clinical Research Center of Diabetes and Metabolic Diseases, Jinan, Shandong 250021, China; ^3^Shandong Key Laboratory of Endocrinology and Lipid Metabolism, Jinan, Shandong 250021, China; ^4^Shandong Prevention and Control Engineering Laboratory of Endocrine and Metabolic Diseases, Jinan, Shandong 250021, China; ^5^Scientific Center, Shandong Provincial Hospital Affiliated to Shandong First Medical University, Jinan, Shandong 250021, China

## Abstract

Nonalcoholic steatohepatitis (NASH) is a liver disease caused by multiple factors, and there is no approved pharmacotherapy. The pathogenesis of NASH remains underexplored. In this study, differentially expressed circular RNAs (circRNAs) were obtained by analyzing NASH-related circRNA datasets, and then, corresponding target microRNAs (miRNAs) and messenger RNAs (mRNAs) were predicted to construct a circRNA–miRNA–mRNA regulatory network. On this basis, a total of 38 circRNAs, 7 miRNAs, and 10 mRNAs were screened out. The present study reveals novel circRNA biomarkers of NASH and reports a potential competing endogenous RNA (ceRNA) regulatory network that might provide insights for further investigation into the underlying pathogenesis of NASH.

## 1. Introduction

Nonalcoholic fatty liver disease (NAFLD) is the most common liver disease worldwide and includes nonalcoholic fatty liver, nonalcoholic steatohepatitis (NASH), and cirrhosis. Nonalcoholic fatty liver (NAFL) is characterized by simple steatosis, whereas NASH is typically characterized by the presence of lobular inflammation and ballooning with or without perisinusoidal fibrosis in addition to steatosis [1]. NAFL is the nonprogressive form of NAFLD, while NASH is the progressive form of NAFLD and may advance to cirrhosis and hepatocellular carcinoma (HCC), which is the leading cause of end-stage liver disease or liver transplantation [2]. The prevalence of NASH has been gradually increasing worldwide in recent years, and worryingly, the liver-specific mortality rate for NASH is high [3]. However, the pathogenesis of NASH has not been fully elucidated.

In recent years, various noncoding RNAs (ncRNAs) acting as competing endogenous RNAs (ceRNAs) have become a major research hotspot for various diseases. MiRNAs and circRNAs are different kinds of ncRNAs [4]. Multiple lines of evidence indicate that other RNAs with miRNA target sites, such as circRNAs, can compete with mRNAs to bind miRNAs [5]. CircRNAs have become a focus of life science and medical research and have been identified as key regulators of many diseases. Studies have shown that circRNAs can act as ceRNAs or miRNA sponges by interacting with miRNAs to sequester these molecules and reduce their regulatory effect on target mRNAs [6]. The circRNA–miRNA–mRNA axis has also been shown to be involved in a variety of cellular events, including apoptosis, vascularization, and metastasis. Studies have shown that the expression profile of circRNA can be a candidate for NASH diagnosis, and the circRNA–miRNA–mRNA pathway may provide clues for studying the pathogenesis of NASH [7, 8]. The exploration of circRNA expression patterns and the circRNA–miRNA–mRNA network in the pathogenesis of NAFLD has gradually been carried out. As the pathogenesis of NASH has not been fully elucidated, it appears to be multifactorial. Moreover, the clinical options for NASH are very limited, and many of the drugs in development have failed in both phase 2 and 3 clinical trials. Therefore, research on NASH still faces great challenges. The role of circRNAs in NASH is a new research field. There are few reports on circRNAs in NASH, so further research is needed. Exploring ncRNAs in NASH may provide useful clues to the pathogenesis of NASH.

Therefore, in this study, bioinformatics methods were used to analyze differentially expressed genes (DEGs) associated with NASH, and then, a ceRNA regulatory network involving circRNA, miRNA, and mRNA was constructed to explore some new circRNAs that might be used as ceRNAs to regulate gene expression in NASH.

## 2. Materials and Methods

### 2.1. Data Collection and Differentially Expressed circRNA (DEC) Identification

The Gene Expression Omnibus (GEO) is a public functional genomics data repository that supports MIAME-compliant data submissions. This database accepts data based on arrays and sequences. Tools are provided to help users query, locate, review, and download research and gene expression profiles [9]. We searched the dataset of NASH in the GEO database, and a series of related microarray datasets that provide circRNA expression profile data in NASH were acquired. We found raw microarray data for the circRNA expression profile GSE134146 and related GPL microarray gene annotation files [10]. DEC data were obtained by analyzing 4 cases of NASH and 4 controls included in the raw files of the GSE134146 dataset. All raw expression data were normalized by log2 transformation. Then, the online analysis tool GEO2R was used to analyze the differences in microarray data, and the DECs of the microarray dataset were determined with *P* < 0.05, Log2-fold change (FC) > 1 or Log2-fold change (FC) < −1 as the criteria.

### 2.2. Prediction of miRNAs

CircInteractome computationally identifies potential binding sites for RNA-binding proteins within circRNAs [11]. It maps RNA-binding protein (RBP) and miRNA-responsive element (MRE) sites on human circRNAs by searching some public databases of circRNAs, miRNAs, and RBPs. It uses the TargetScan prediction tool to predict miRNAs that may target circRNAs. miRNet is an easy-to-use web-based tool designed to create, customize, visually explore, and functionally interpret miRNA target interaction networks. It can be integrated into a powerful network visualization system by integrating multiple high-quality miRNA target data sources and advanced statistical methods [12]. The relevant target miRNAs of these selected DECs were predicted using two network tools, miRNet and CircInteractome. Overlapping miRNAs for both algorithms were predicted as potential target miRNAs for DECs.

The expression dataset GSE33857 of NASH-related miRNAs was retrieved from the GEO database and includes information for 7 NASH cases and 12 controls. The GSE33857 chip was analyzed by GEO2R, and the differentially expressed miRNAs that overlapped with the predicted targets were included in the next analysis.

### 2.3. Forecasting of miRNA-Targeted Genes

miRWalk is a comprehensive miRNA target gene database that includes the miRNA target gene information of humans, mice, rats, dogs, cows, and other species. It not only includes the full-length gene sequence record of the complete miRNA-binding site but is also compatible with the 12 existing miRNA target prediction programs (DIANA-microTv4.0, DIANA-microT-CDS, miRanda-rel2010, mirBridge, miRDB4.0, miRmap, miRNAMap, doRiNA, i.e., PicTar2, PITA RNA22v2, RNAhybrid2.1 and Targetscan6.2). This database can be used to predict the associated combined information set. The two databases miRWalk and miRNet were used to predict target mRNAs of differentially expressed miRNAs. Then, the overlapping DEGs were selected. The GSE24807 dataset is related to the gene expression profiles of NASH, and the raw data were also analyzed with GEO2R [13, 14]. Only the DEGs obtained by intersecting the genes of the dataset with the predicted target genes were included in the ceRNA network.

### 2.4. Functional Enrichment Analysis of Overlapping Genes and Establishment of the Protein–Protein Interaction (PPI) Network

The Database for Annotation, Visualization, and Integrated Discovery (DAVID) provides a comprehensive set of functional annotation tools for investigators to understand the biological meaning behind large lists of genes [15]. It was used to perform Gene Ontology (GO) analysis and Kyoto Encyclopedia of Genes and Genomes (KEGG) pathway enrichment analysis. The Search Tool for the Retrieval of Interacting Genes database (STRING) provides credible information on interactions between proteins and supplies detailed annotation [16].

### 2.5. Construction of circRNA–miRNA–mRNA Network

To reveal the relationships among circRNAs, miRNAs, and mRNAs, a circRNA–miRNA–mRNA network was constructed by combining circRNA–miRNA interactions with miRNA–mRNA interactions using Cytoscape.

## 3. Results

### 3.1. Identification of DECs in NASH

**Figure 1:**
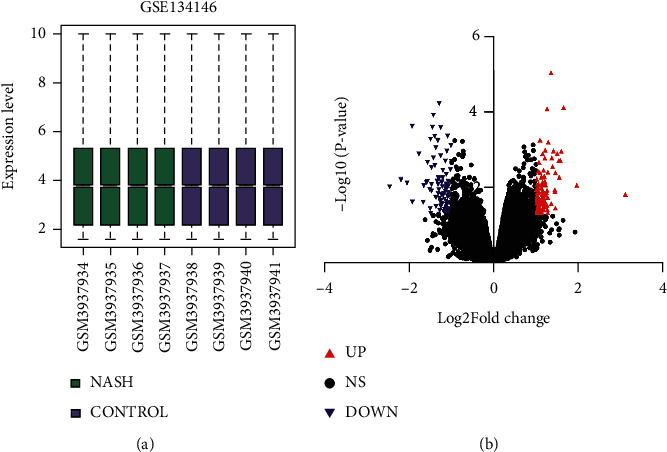
(a) Sample expression correction box diagram. Analysis of normalized GSE134146 data. (b) Volcano diagram of differentially expressed genes. The red dots represent significantly upregulated genes, and the blue dots represent significantly downregulated genes. Log FC > 1 or Log FC < −1, and *P* < 0.05.

**Figure 2:**
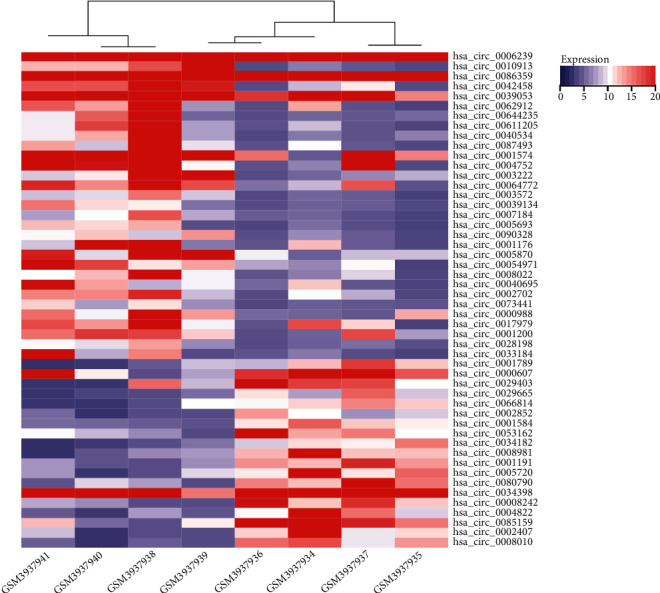
Sample clustering diagram. These are the 50 most significant differentially expressed circRNAs in GSE134146, and the change in color represents the difference in expression. Blue represents low expression; red represents high expression.

To construct the interaction network between circRNAs and miRNAs in NASH, DECs should be determined first. A microarray dataset GSE134146 was obtained from the GEO database, which includes 4 NASH cases and 4 controls. The gene chip was from the Agilent-074301 Arraystar Human CircRNA microarray V2 platform. The online analysis tool GEO2R was used to analyze a series of differentiated circRNAs, such as boxplots (Figure 1(a)). *P* < 0.05 and |logFC| > 1 were used as the screening criteria in the GSE134146 dataset. There were 192 DECs, including 96 upregulated circRNAs and 96 downregulated circRNAs, between NASH patients and controls (Figure 1(b)). The expression differences of the top 50 circRNAs with the most significant differences in 4 NASH tissues and 4 control tissues are shown in Figure 2.

### 3.2. Identification of 54 circRNA–miRNA Interactions

Growing evidence indicates that circRNAs regulate gene expression via miRNA sponges. Therefore, some miRNAs related to the DECs we obtained were predicted based on this ceRNA theory. We collected and explored their potential miRNAs through the CircInteractome and miRNet online databases. Among them, 603 miRNAs were found in CircInteractome, and 640 miRNAs were found in miRNet. To ensure accuracy, we used the intersection of the two to obtain 58 overlapping predicted miRNAs. The gene chip dataset GSE33857 from the GEO database was used to verify the predicted miRNAs, and 8 miRNAs that interacted with circRNAs were obtained. According to the related regulatory relationship between circRNA–miRNAs, only 39 circRNAs (25 upregulated, 14 downregulated) and 8 miRNAs (3 upregulated, 5 downregulated) were included in the ceRNA network study. A total of 54 circRNA–miRNA interactions were identified (Table 1).

### 3.3. Analysis of miRNA–mRNA Interactions

**Figure 3:**
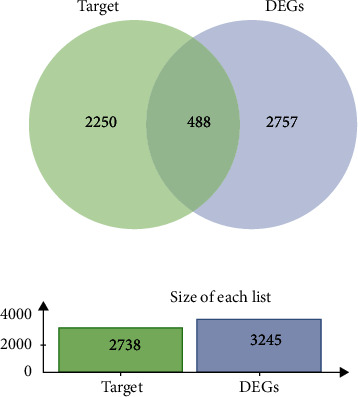
Venn diagram of overlapping DEGs. Four hundred and eighty-eight overlapping genes were obtained by crossing miRNA-targeted genes and DEGs from the GEO database.

We obtained 8 miRNAs associated with circRNAs. To explore the functions of these miRNAs in NASH, we used two databases, miRWalk and miRNet, to predict miRNA-related target genes. A total of 2738 folded predicted target genes were found in both databases. The GSE24807 dataset from the GEO database was used to verify the DEGs. A total of 3245 DEGs were obtained from the dataset. In addition, as shown in Figures 3, 448 overlapping genes were identified by intersecting miRNA target genes with DEGs in GEO. Based on the regulatory relationship between miRNAs and mRNAs, 291 genes were included in the list of ceRNAs. A circRNA–miRNA–mRNA regulatory network was constructed by using Cytoscape software (Table 1).

### 3.4. Functional Enrichment Analyses and PPI Network Construction

**Figure 4:**
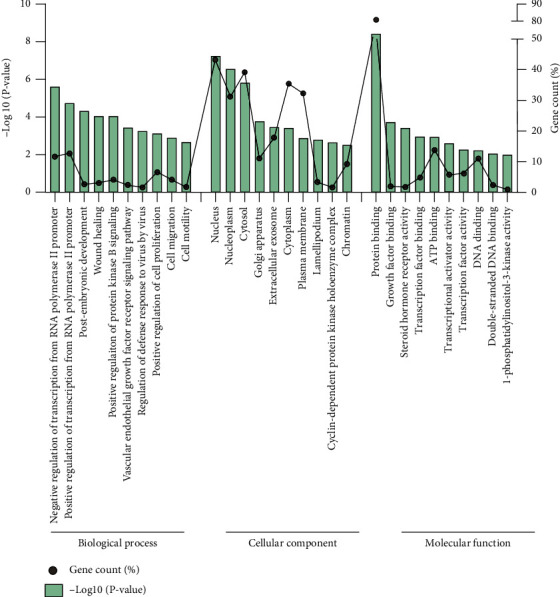
Functional enrichment analysis of 291 DEGs. The enriched Gene Ontology (GO) terms fell into three main GO categories: BP: biological process; CC: cellular component; MF: molecular function.

**Figure 5:**
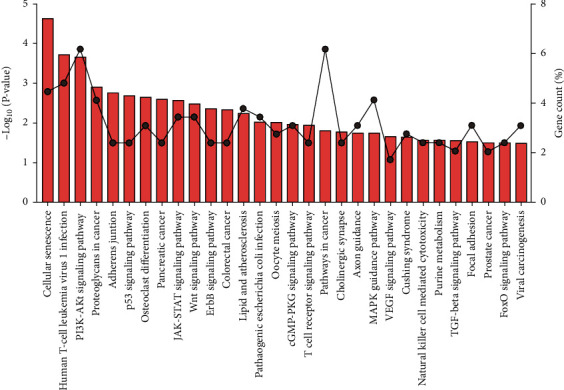
KEGG analysis. KEGG enrichment pathway analysis was performed on 291 differentially expressed genes in the ceRNA network, and the top 30 pathways were visualized. The bars represent *P* values, and the dots represent the percentages of genes included in the process among the 291 genes.

Terms related to the DEGs were divided into three functional groups, including biological processes (BP), molecular functions (MF), and cell compositions (CC), using DAVID. The values of the individual components in the GO analysis are shown in Figure 4. In the BP category, 291 DEGs were mainly involved in negative regulation of transcription from RNA polymerase II promoter, positive regulation of transcription from RNA polymerase II promoter, postembryonic development, wound healing, positive regulation of protein kinase B signaling, vascular endothelial growth factor receptor signaling pathway, regulation of defense response to virus by virus, positive regulation of cell proliferation, cell migration, cell motility, and other processes. In the MF category, the genes were mainly enriched in protein binding, growth factor binding, steroid hormone receptor activity, transcription factor binding, ATP binding, transcriptional activator activity, transcription factor activity, sequence-specific DNA binding, DNA binding, double-stranded DNA binding, 1-phosphatidylinositol-3-kinase activity, etc. In the CC category, the genes were mainly enriched in the nucleus, nucleoplasm, cytosol, Golgi apparatus, extracellular exosome, cytoplasm, plasma membrane, lamellipodium, cyclin-dependent protein kinase holoenzyme complex, chromatin, etc. KEGG signaling pathway showed that genes were mainly enriched in cellular senescence, human T cell leukemia virus 1 infection, PI3K-Akt signaling pathway, proteoglycans in cancer, adherens junction, p53 signaling pathway, osteoclast differentiation, pancreatic cancer, JAK-STAT signaling pathway, Wnt signaling pathway, ErbB signaling pathway, colorectal cancer, lipid and atherosclerosis, pathogenic Escherichia coli infection, oocyte meiosis, cGMP-PKG signaling pathway, T cell receptor signaling pathway, pathways in cancer, cholinergic synapse, axon guidance, MAPK signaling pathway, VEGF signaling pathway, Cushing syndrome, natural killer cell-mediated cytotoxicity, purine metabolism, TGF-beta signaling pathway, focal adhesion, prostate cancer, FoxO signaling pathway, viral carcinogenesis, etc. The results in the KEGG analysis are shown in Figure 5.

### 3.5. Construction of a circRNA–miRNA–mRNA Network

**Figure 6:**
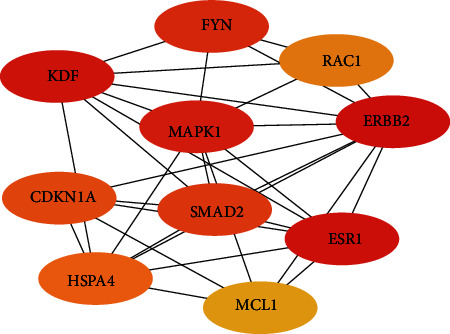
Hub genes in the NASH-associated PPI network. The 10 hub genes were identified from the PPI network using cytoHubba. The line indicates an interaction between two genes.

**Figure 7:**
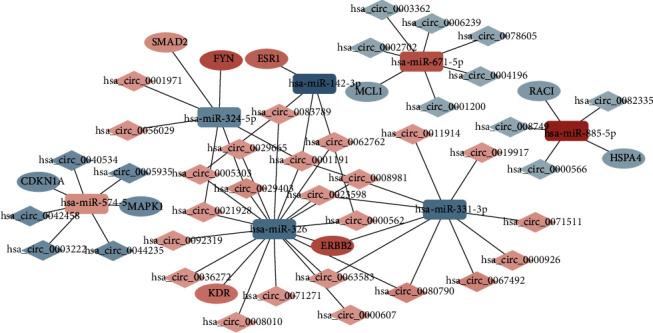
NASH-related circRNA–miRNA–hub gene axis. The key NASH-related circRNA, miRNA, and hub gene axis were composed of 38 circRNAs, 7 miRNAs, and 10 hub genes. Ovals indicate mRNAs, rectangles indicate miRNAs, and diamonds indicate circRNAs. Red indicates upregulation, and blue indicates downregulation. The shade of color indicates the degree of up or down.

To further explore the effect of the circRNA–miRNA regulatory network on the expression levels of NASH genes, a PPI network was constructed, and 558 pairs of genes with interactions were found through the STRING database. The PPI network was imported into Cytoscape, and the cytoHubba plug-in was used to further screen hub genes according to the maximal clique centrality (MCC) algorithm. Then, a subnetwork with 10 nodes and 31 edges was selected, which revealed the critical roles of the ten genes (KDR, FYN, RAC1, MAPK1, ERBB2, CDKN1A, HSPA4, SMAD2, MCL1, and ESR1) in NASH (Figure 6). According to the negative regulatory relationship between ceRNAs, a total of 10 genes and miRNAs were included in the network. After this, a network about the association among these circRNA, miRNAs, and hub genes was built (Figure 7). It provided a visualization of the connections among the 38 DECs (hsa_circ_0000566, hsa_circ_0087493, hsa_circ_0082335, hsa_circ_0004196, hsa_circ_0002702, hsa_circ_0003362, hsa_circ_0006239, hsa_circ_0078605, hsa_circ_0001200, hsa_circ_0044235, hsa_circ_0042458, hsa_circ_0040534, hsa_circ_0003222, hsa_circ_0005935, hsa_circ_0000562, hsa_circ_0005303, hsa_circ_0000607, hsa_circ_0036272, hsa_circ_0029403, hsa_circ_0062762, hsa_circ_0080790, hsa_circ_0001191, hsa_circ_0008010, hsa_circ_0023598, hsa_circ_0092319, hsa_circ_0071271, hsa_circ_0008981, hsa_circ_0021928, hsa_circ_0029665, hsa_circ_0083789, hsa_circ_0063583, hsa_circ_0071511, hsa_circ_0011914, hsa_circ_0019917, hsa_circ_0067492, hsa_circ_0000926, hsa_circ_0001971, hsa_circ_0056029), 7 miRNAs (hsa-miR-326, hsa-miR-324-5p, hsa-miR-885-5p, hsa-miR-574-5p, hsa-miR-671 -5p, hsa-miR-142-3p, and hsa-miR-331-3p) and 10 hub genes (KDR, FYN, RAC1, MAPK1, ERBB2, CDKN1A, HSPA4, SMAD2, MCL1, and ESR1).

## 4. Discussion

We successfully constructed a circRNA-related ceRNA regulatory network by integrating and analyzing the expression differences of NASH-related circRNAs, miRNAs, and mRNAs in the GSE134146, GSE33857, and GSE24807 datasets in the GEO database. We found that 39 circRNAs may indirectly regulate 291 mRNAs (or genes) through competitive binding with 8 miRNAs. Among the regulated genes, the 10 most critical central genes were screened out. Then, a network of circRNAs, miRNAs, and hub genes was constructed, which contained 38 differentially expressed circRNAs, 7 miRNAs, and 10 hub genes. These abnormally expressed ceRNAs in NASH have the potential to be excellent biomarkers.

The importance of NASH is self-evident, as it may promote the occurrence and development of HCC. NAFLD is a pathological manifestation of metabolic syndrome in the liver. Specifically, it is a form of hepatic steatosis caused by accumulation of liver fat and is closely related to metabolic disorders such as obesity, type 2 diabetes, insulin resistance, hypertension, and hyperlipidemia. NASH is the progressive form of NAFLD. Around 20%-27% of the NAFLD patients develop NASH [17]. NASH is characterized by hepatic steatosis, inflammation, hepatocyte damage, and fibrosis, with inflammation playing a key role in its progression. Liver inflammation is a critical factor in the transition from NAFLD to NASH. Therefore, inflammation is a key pathophysiological mechanism of NASH and a target for therapeutic intervention. The KEGG pathway enrichment results in the current study showed that 291 DEGs were mainly involved in the PI3K-Akt signaling pathway, JAK-STAT signaling pathway, Wnt signaling pathway, cGMP-PKG signaling pathway, T cell receptor signaling pathway, MAPK signaling pathway, VEGF signaling pathway, etc. Most of these pathways are classical pathways related to inflammation and lipid metabolism.

After multiple screenings, a total of 10 hub genes related to NASH (KDR, FYN, RAC1, MAPK1, ERBB2, CDKN1A, HSPA4, SMAD2, MCL1, and ESR1) in the circRNA–miRNA–mRNA network were identified. Some of them have been linked to liver-related diseases. For example, Zheng et al. identified CDKN1A as a potential key regulator of NASH via dynamic network analysis and dynamic gene coexpression module analysis [18]. Furthermore, studies have shown that the circRNA MAN2B2 promotes the proliferation of hepatoma cells through the miRNA-217/MAPK1 axis [19], which indirectly supports our results. Other studies have shown that HSPA4 is significantly correlated with the prognosis and immune regulation of HCC. Therefore, HSPA4 might be a potential diagnostic and prognostic biomarker as well as a therapeutic target for HCC [20]. In previous studies, these genes, including both MAPK1 and HSPA4, were not reported to be related to NASH but other liver-related diseases. By analyzing the biological processes of these DEGs, we found that these genes may also play an important role in the pathogenesis of NASH.

The importance of ceRNAs in various diseases is emerging, and some ceRNAs have been found to be associated with NASH. There is also evidence that indicates the important regulatory role of miRNAs in NASH [21, 22]. Potential targets of differentially expressed miRNAs were known to play a role in lipid metabolism, cell growth and differentiation, apoptosis, and inflammation. For example, overexpression of miR-142-5p inhibits the progression of NASH by targeting thymic stromal lymphopoietin and inhibiting the JAK-STAT signaling pathway. Thus, miR-142-5p might be a novel latent target for NASH therapy [23]. This is consistent with our findings. In addition, miRNA-223 ameliorates NASH by targeting multiple inflammatory genes in hepatocytes [24]. The role for miR-296 is to regulate lipoapoptosis by targeting p53 upregulated modulator of apoptosis. Hepatocyte lipoapoptosis is a key mediator of liver injury in NASH [25], which makes our data more convincing. Emerging studies seem to establish miRNAs as excellent noninvasive tools for the early diagnosis and treatment of various stages of liver diseases [26]. Recent studies suggest that circRNA may be involved in the pathogenesis of NASH [27]. For instance, steatohepatitis-associated circRNA ATP5B regulator, a mitochondria-located circRNA, was demonstrated to play an important role in alleviating NASH by reducing mROS output [10]. In addition, antagonizing the circRNA_002581-miR-122-CPEB1 axis could alleviate NASH by restoring the PTEN-AMPK-mTOR pathway [8]. However, to date, there is no authoritative agency-approved therapeutic drug on the market. The complex pathogenesis, disease heterogeneity, diagnostic barriers, and selection of treatment endpoints also bring great challenges to NASH research. Therefore, research on NASH still has a long way to go.

Certain potential limitations existed in our study. Further evidence from both in vivo and in vitro experiments is needed for verification. Further study on the physiopathologic mechanism of NASH is being performed on the basis of the current bioinformatics analysis.

In this study, bioinformatics methods were used to integrate NASH and normal liver tissue gene chips to screen out DECs and then search for corresponding miRNAs and competing mRNAs to provide a reference for further research on the pathogenesis of NASH. These circRNAs, miRNAs, and mRNAs were found to be abnormally expressed in NASH, and they have the potential to be potential biomarkers for NASH screening. They also have the potential to enter routine clinical practice and be used as predictive markers of the response to NASH-targeted therapies.

## 5. Conclusions

In this study, we constructed a NASH-related ceRNA network by integrating and analyzing the expression differences of NASH-related circRNAs, miRNAs, and mRNAs in the GEO database. These differentially expressed ceRNAs have the potential to be biomarkers for NASH screening and may provide valuable clues for further research on the pathogenesis of NASH.

## Figures and Tables

**Table 1:** NASH-related ceRNA regulatory network.

ceRNA regulatory network		ceRNA regulatory network	
hsa_circ_0000566	hsa-miR-885-5p	hsa-miR-142-3p	LOX
hsa_circ_0087493	hsa-miR-885-5p	hsa-miR-142-3p	BTNL9
hsa_circ_0082335	hsa-miR-885-5p	hsa-miR-142-3p	NUDT8
hsa_circ_0004196	hsa-miR-671-5p	hsa-miR-142-3p	NIN
hsa_circ_0002702	hsa-miR-671-5p	hsa-miR-142-3p	DUSP7
hsa_circ_0003362	hsa-miR-671-5p	hsa-miR-142-3p	PIK3CG
hsa_circ_0006239	hsa-miR-671-5p	hsa-miR-142-3p	XRCC1
hsa_circ_0078605	hsa-miR-671-5p	hsa-miR-142-3p	NCKAP1
hsa_circ_0001200	hsa-miR-671-5p	hsa-miR-142-3p	MMD
hsa_circ_0044235	hsa-miR-574-5p	hsa-miR-142-3p	AP4B1
hsa_circ_0042458	hsa-miR-574-5p	hsa-miR-142-3p	VKORC1
hsa_circ_0040534	hsa-miR-574-5p	hsa-miR-142-3p	CTTN
hsa_circ_0003222	hsa-miR-574-5p	hsa-miR-142-3p	KLHL24
hsa_circ_0005935	hsa-miR-574-5p	hsa-miR-142-3p	POLI
hsa_circ_0000562	hsa-miR-326	hsa-miR-142-3p	ATF5
hsa_circ_0005303	hsa-miR-326	hsa-miR-142-3p	ABCG2
hsa_circ_0000607	hsa-miR-326	hsa-miR-142-3p	CHST11
hsa_circ_0036272	hsa-miR-326	hsa-miR-142-5p	CD109
hsa_circ_0029403	hsa-miR-326	hsa-miR-142-5p	RMND5A
hsa_circ_0062762	hsa-miR-326	hsa-miR-142-5p	UHMK1
hsa_circ_0080790	hsa-miR-326	hsa-miR-142-5p	PCBP2
hsa_circ_0001191	hsa-miR-326	hsa-miR-142-5p	NFIB
hsa_circ_0008010	hsa-miR-326	hsa-miR-142-5p	RAB34
hsa_circ_0023598	hsa-miR-326	hsa-miR-142-5p	UBXN2A
hsa_circ_0092319	hsa-miR-326	hsa-miR-142-5p	LAPTM4A
hsa_circ_0071271	hsa-miR-326	hsa-miR-142-5p	KLHDC10
hsa_circ_0008981	hsa-miR-326	hsa-miR-142-5p	ZBTB20
hsa_circ_0021928	hsa-miR-326	hsa-miR-142-5p	PLEK
hsa_circ_0029665	hsa-miR-326	hsa-miR-142-5p	SCD
hsa_circ_0083789	hsa-miR-326	hsa-miR-142-5p	UBE2H
hsa_circ_0063583	hsa-miR-326	hsa-miR-142-5p	ZFP36L1
hsa_circ_0071511	hsa-miR-331-3p	hsa-miR-142-5p	VAPA
hsa_circ_0011914	hsa-miR-331-3p	hsa-miR-142-5p	ZNF248
hsa_circ_0080790	hsa-miR-331-3p	hsa-miR-142-5p	SH2B3
hsa_circ_0019917	hsa-miR-331-3p	hsa-miR-142-5p	TMEM98
hsa_circ_0067492	hsa-miR-331-3p	hsa-miR-142-5p	ZNF585B
hsa_circ_0001191	hsa-miR-331-3p	hsa-miR-142-5p	RHOC
hsa_circ_0023598	hsa-miR-331-3p	hsa-miR-142-5p	MYO1D
hsa_circ_0000926	hsa-miR-331-3p	hsa-miR-142-5p	ZNF614
hsa_circ_0063583	hsa-miR-331-3p	hsa-miR-142-5p	TAOK1
hsa_circ_0001971	hsa-miR-324-5p	hsa-miR-142-5p	TRIM66
hsa_circ_0029403	hsa-miR-324-5p	hsa-miR-142-5p	EEF1A1
hsa_circ_0001191	hsa-miR-324-5p	hsa-miR-142-5p	ARF6
hsa_circ_0056029	hsa-miR-324-5p	hsa-miR-142-5p	ATXN1
hsa_circ_0021928	hsa-miR-324-5p	hsa-miR-142-5p	SOX5
hsa_circ_0000562	hsa-miR-142-3p	hsa-miR-142-5p	IGF2
hsa_circ_0005303	hsa-miR-142-3p	hsa-miR-142-5p	APOL6
hsa_circ_0001191	hsa-miR-142-3p	hsa-miR-142-5p	C1orf21
hsa_circ_0000562	hsa-miR-142-5p	hsa-miR-142-5p	FAM126B
hsa_circ_0005303	hsa-miR-142-5p	hsa-miR-142-5p	GABPB1
hsa_circ_0011898	hsa-miR-142-5p	hsa-miR-142-5p	STMN1
hsa_circ_0029403	hsa-miR-142-5p	hsa-miR-142-5p	MTHFD2
hsa_circ_0001191	hsa-miR-142-5p	hsa-miR-142-5p	CCNE1
hsa_circ_0063583	hsa-miR-142-5p	hsa-miR-142-5p	GXYLT1
hsa-miR-574-5p	ITPRIP	hsa-miR-142-5p	NAGK
hsa-miR-574-5p	EIF5	hsa-miR-142-5p	DENND4C
hsa-miR-574-5p	OSMR	hsa-miR-142-5p	FIGN
hsa-miR-574-5p	ZNF738	hsa-miR-142-5p	MIB1
hsa-miR-574-5p	LPP	hsa-miR-142-5p	PIK3C2A
hsa-miR-574-5p	RREB1	hsa-miR-142-5p	HELZ
hsa-miR-574-5p	FHL2	hsa-miR-142-5p	C5orf15
hsa-miR-574-5p	MAPK1	hsa-miR-142-5p	PLS1
hsa-miR-574-5p	ETV6	hsa-miR-142-5p	CREBRF
hsa-miR-574-5p	CD28	hsa-miR-142-5p	TNFSF13B
hsa-miR-574-5p	CD164	hsa-miR-142-5p	GPR65
hsa-miR-574-5p	ATP2B1	hsa-miR-324-5p	SMAD2
hsa-miR-574-5p	SLC7A2	hsa-miR-324-5p	RAPH1
hsa-miR-574-5p	SLITRK3	hsa-miR-324-5p	PCDH9
hsa-miR-574-5p	WAC	hsa-miR-324-5p	NUFIP2
hsa-miR-574-5p	AMER1	hsa-miR-324-5p	RMND5A
hsa-miR-574-5p	RNF152	hsa-miR-324-5p	CCND3
hsa-miR-574-5p	ASH1L	hsa-miR-324-5p	TGIF1
hsa-miR-574-5p	CDKN1A	hsa-miR-324-5p	PRCP
hsa-miR-574-5p	C12orf60	hsa-miR-324-5p	HNRNPDL
hsa-miR-574-5p	NCDN	hsa-miR-324-5p	FYN
hsa-miR-574-5p	SH3TC2	hsa-miR-324-5p	AJUBA
hsa-miR-574-5p	ACVR2B	hsa-miR-324-5p	PPP3CA
hsa-miR-574-5p	APBA1	hsa-miR-324-5p	SOBP
hsa-miR-574-5p	RAB3IP	hsa-miR-324-5p	CTTNBP2NL
hsa-miR-574-5p	VPS36	hsa-miR-324-5p	NDFIP2
hsa-miR-574-5p	HP1BP3	hsa-miR-324-5p	COL14A1
hsa-miR-574-5p	HIPK2	hsa-miR-324-5p	SCD
hsa-miR-574-5p	CLCF1	hsa-miR-324-5p	ETS1
hsa-miR-574-5p	PPP2R1B	hsa-miR-324-5p	WASF2
hsa-miR-574-5p	ZNF621	hsa-miR-324-5p	AP1S2
hsa-miR-671-5p	FMNL3	hsa-miR-324-5p	CCND2
hsa-miR-671-5p	SURF4	hsa-miR-324-5p	NFATC1
hsa-miR-671-5p	SMC1A	hsa-miR-324-5p	DBNL
hsa-miR-671-5p	TBCEL	hsa-miR-324-5p	ZBTB44
hsa-miR-671-5p	IL15RA	hsa-miR-324-5p	TMEM63B
hsa-miR-671-5p	NRG1	hsa-miR-324-5p	NELFB
hsa-miR-671-5p	OSMR	hsa-miR-324-5p	ZNF747
hsa-miR-671-5p	SOD2	hsa-miR-324-5p	EFNB1
hsa-miR-671-5p	WBP2	hsa-miR-324-5p	PBX1
hsa-miR-671-5p	ATF3	hsa-miR-324-5p	UBE2I
hsa-miR-671-5p	RREB1	hsa-miR-324-5p	PDRG1
hsa-miR-671-5p	ODC1	hsa-miR-324-5p	ST3GAL1
hsa-miR-671-5p	ERP29	hsa-miR-324-5p	NME3
hsa-miR-671-5p	CPEB3	hsa-miR-324-5p	GXYLT1
hsa-miR-671-5p	WDR43	hsa-miR-324-5p	DBT
hsa-miR-671-5p	FEM1B	hsa-miR-324-5p	AK3
hsa-miR-671-5p	FNDC3B	hsa-miR-324-5p	CBX3
hsa-miR-671-5p	MRM1	hsa-miR-324-5p	ERLIN2
hsa-miR-671-5p	TC2N	hsa-miR-324-5p	FAT3
hsa-miR-671-5p	SSR1	hsa-miR-324-5p	MMD
hsa-miR-671-5p	TBRG1	hsa-miR-324-5p	NFIX
hsa-miR-671-5p	TNPO1	hsa-miR-324-5p	MGMT
hsa-miR-671-5p	LNPEP	hsa-miR-326	PAQR8
hsa-miR-671-5p	GNS	hsa-miR-326	NUFIP2
hsa-miR-671-5p	NSF	hsa-miR-326	RMND5A
hsa-miR-671-5p	NR4A3	hsa-miR-326	PTPA
hsa-miR-671-5p	MCTS1	hsa-miR-326	SLC39A1
hsa-miR-671-5p	NSUN5	hsa-miR-326	PDE1B
hsa-miR-671-5p	ABHD2	hsa-miR-326	SYNE2
hsa-miR-671-5p	PRKAR1A	hsa-miR-326	SPG7
hsa-miR-671-5p	ANGEL1	hsa-miR-326	KLHDC10
hsa-miR-671-5p	MCL1	hsa-miR-326	SDC1
hsa-miR-671-5p	NAMPT	hsa-miR-326	CCND2
hsa-miR-671-5p	KLHL7	hsa-miR-326	SMPD1
hsa-miR-671-5p	LDLR	hsa-miR-326	ERBB2
hsa-miR-671-5p	RAB3IP	hsa-miR-326	TBL1XR1
hsa-miR-671-5p	HP1BP3	hsa-miR-326	ZBTB4
hsa-miR-671-5p	SERINC3	hsa-miR-326	TAOK1
hsa-miR-671-5p	INSR	hsa-miR-326	CLU
hsa-miR-671-5p	SAA2	hsa-miR-326	GPC4
hsa-miR-671-5p	DDX21	hsa-miR-326	IL10RA
hsa-miR-671-5p	CHI3L1	hsa-miR-326	KDR
hsa-miR-671-5p	NHLRC2	hsa-miR-326	MTHFD2
hsa-miR-885-5p	TBCEL	hsa-miR-326	RECQL5
hsa-miR-885-5p	SOD2	hsa-miR-326	FPR1
hsa-miR-885-5p	ODC1	hsa-miR-326	NREP
hsa-miR-885-5p	CPEB3	hsa-miR-326	PHKA1
hsa-miR-885-5p	RAC1	hsa-miR-326	DUSP7
hsa-miR-885-5p	CD164	hsa-miR-326	ERLIN2
hsa-miR-885-5p	KLF6	hsa-miR-326	PDE3A
hsa-miR-885-5p	TGFBR1	hsa-miR-326	ABCC6
hsa-miR-885-5p	POU2F1	hsa-miR-326	VKORC1
hsa-miR-885-5p	GOLT1B	hsa-miR-326	ARRDC1
hsa-miR-885-5p	HSPA4	hsa-miR-326	LRRC75B
hsa-miR-885-5p	SRSF1	hsa-miR-326	UBE2Z
hsa-miR-885-5p	WDR36	hsa-miR-326	SLC47A1
hsa-miR-885-5p	SULT1B1	hsa-miR-326	FSCN1
hsa-miR-885-5p	CHD8	hsa-miR-331-3p	SRGAP1
hsa-miR-885-5p	ACSS1	hsa-miR-331-3p	NUFIP2
hsa-miR-885-5p	TMC7	hsa-miR-331-3p	PCBP2
hsa-miR-142-3p	AFF1	hsa-miR-331-3p	NFIB
hsa-miR-142-3p	ST6GAL1	hsa-miR-331-3p	QKI
hsa-miR-142-3p	KLF13	hsa-miR-331-3p	PRICKLE1
hsa-miR-142-3p	HECW2	hsa-miR-331-3p	CTDSP1
hsa-miR-142-3p	SRGAP1	hsa-miR-331-3p	AFAP1
hsa-miR-142-3p	NUFIP2	hsa-miR-331-3p	HIC2
hsa-miR-142-3p	RMND5A	hsa-miR-331-3p	SDC1
hsa-miR-142-3p	CLCN5	hsa-miR-331-3p	SCD
hsa-miR-142-3p	CHID1	hsa-miR-331-3p	ETS1
hsa-miR-142-3p	AK4	hsa-miR-331-3p	PITPNA
hsa-miR-142-3p	NFIB	hsa-miR-331-3p	PTPRT
hsa-miR-142-3p	C11orf54	hsa-miR-331-3p	ZBTB38
hsa-miR-142-3p	CREB5	hsa-miR-331-3p	ENTPD1
hsa-miR-142-3p	CCNG1	hsa-miR-331-3p	AP2B1
hsa-miR-142-3p	FAM102A	hsa-miR-331-3p	ZFP36L1
hsa-miR-142-3p	EI24	hsa-miR-331-3p	GUCD1
hsa-miR-142-3p	ZBTB20	hsa-miR-331-3p	SMG6
hsa-miR-142-3p	RGS5	hsa-miR-331-3p	ERBB2
hsa-miR-142-3p	SCD	hsa-miR-331-3p	FBXO44
hsa-miR-142-3p	CLIC4	hsa-miR-331-3p	GCK
hsa-miR-142-3p	ENTPD1	hsa-miR-331-3p	MPLKIP
hsa-miR-142-3p	NR2C1	hsa-miR-331-3p	NRP2
hsa-miR-142-3p	RABGAP1L	hsa-miR-331-3p	PMPCB
hsa-miR-142-3p	ZFP36L1	hsa-miR-331-3p	EXOC6B
hsa-miR-142-3p	VAPA	hsa-miR-331-3p	ZBTB4
hsa-miR-142-3p	CIITA	hsa-miR-331-3p	NUCKS1
hsa-miR-142-3p	CLIC2	hsa-miR-331-3p	TAOK1
hsa-miR-142-3p	SERPINA4	hsa-miR-331-3p	PIK3R3
hsa-miR-142-3p	IFNAR2	hsa-miR-331-3p	MECP2
hsa-miR-142-3p	MPLKIP	hsa-miR-331-3p	ZER1
hsa-miR-142-3p	CUX1	hsa-miR-331-3p	ARSD
hsa-miR-142-3p	RBMS1	hsa-miR-331-3p	IGFBP5
hsa-miR-142-3p	NFIA	hsa-miR-331-3p	RNF146
hsa-miR-142-3p	NELFB	hsa-miR-331-3p	IGF2
hsa-miR-142-3p	TFB1M	hsa-miR-331-3p	APOL6
hsa-miR-142-3p	SLC30A6	hsa-miR-331-3p	FAM126B
hsa-miR-142-3p	NUCKS1	hsa-miR-331-3p	PMAIP1
hsa-miR-142-3p	TAOK1	hsa-miR-331-3p	COTL1
hsa-miR-142-3p	TOB1	hsa-miR-331-3p	ADAMTS5
hsa-miR-142-3p	SF3A1	hsa-miR-331-3p	KREMEN1
hsa-miR-142-3p	ESR1	hsa-miR-331-3p	TMEM254
hsa-miR-142-3p	PBX1	hsa-miR-331-3p	CLDN10
hsa-miR-142-3p	EEF1A1	hsa-miR-331-3p	ADCY1
hsa-miR-142-3p	GPC4	hsa-miR-331-3p	FAT3
hsa-miR-142-3p	ATXN1	hsa-miR-331-3p	EIF2S3
hsa-miR-142-3p	NCK2	hsa-miR-331-3p	LZTS2
hsa-miR-142-3p	ZMYND8	hsa-miR-331-3p	COL6A2
hsa-miR-142-3p	ZNF473	hsa-miR-331-3p	CAMK2G
hsa-miR-142-3p	HMGB1	hsa-miR-331-3p	RRP1B
hsa-miR-142-3p	PIAS2	hsa-miR-331-3p	CDIPT
hsa-miR-142-3p	DPY19L4	hsa-miR-331-3p	WDR33
hsa-miR-142-3p	KRIT1	hsa-miR-331-3p	CREBRF
hsa-miR-142-3p	FBXO7	hsa-miR-331-3p	CD248
hsa-miR-142-3p	CCNE1	hsa-miR-331-3p	ZDHHC8
hsa-miR-142-3p	CCDC28B	hsa-miR-331-3p	HIC1

## Data Availability

All data generated or analyzed in this study are contained in the GEO database and this published article.
